# Rethinking Youth Mental Health: Emerging Risks, Transdiagnostic Dimensions, and New Therapeutic Approaches

**DOI:** 10.3390/brainsci16101096

**Published:** 2026-10-12

**Authors:** Ilaria Riboldi, Giuseppe Carrà

**Affiliations:** School of Medicine and Surgery, University of Milano-Bicocca, Via Cadore 48, 20900 Monza, Italy

## 1. Introduction

Mental disorders among children, adolescents, and young adults represent a major and growing public health concern [1]. Indeed, these developmental stages are characterised by profound neurobiological, psychological, and social changes that may increase vulnerability to psychopathology, particularly through their interaction with evolving environmental and sociocultural conditions [2]. Such vulnerability may therefore be conceptualised as emerging from the dynamic interplay between individual characteristics and contextual influences, rather than from isolated risk factors [2]. This complexity thus supports a broader, transdiagnostic conceptualisation of youth mental health, in which shared processes may contribute to different psychopathological trajectories and provide clinically relevant targets for prevention and intervention [3].

In addition, the social environments in which young people develop have been profoundly transformed by digital technologies [4]. Social media, online communities, and digitally mediated interactions are now embedded in everyday life and may constitute both sources of vulnerability and potential instruments for prevention and mental health promotion [5]. Within this context, the contributions to the Special Issue *Emerging Trends in Youth Mental Health* examine developmental and interpersonal vulnerabilities, digital influences, clinical heterogeneity, and emerging approaches to prevention and treatment across different stages of youth development.

## 2. Developmental, Social, and Digital Dimensions of Youth Mental Health

A first theme emerging from this Special Issue concerns the interplay among social connectedness, traumatic experience, and psychological vulnerability in young populations (Contribution 1). Indeed, evidence from university students in Italy and the UK suggests that loneliness may act as a relevant psychosocial mechanism linking post-traumatic stress symptoms with anxiety and depression. More specifically, loneliness was found to partially mediate these associations, accounting for approximately 22% of the relationship with anxiety symptoms and 33% of that with depressive symptoms (Contribution 1). Consistently, such findings support the broader hypothesis that diminished social connectedness may represent one of the pathways through which exposure to stressful or potentially traumatic experiences contributes to subsequent psychological distress in early adulthood [6]. This mechanism may be particularly salient in university contexts, where relocation, changes in interpersonal networks, increasing academic demands, and the transition toward greater autonomy may simultaneously intensify stress exposure and weaken perceived social support [7]. Accordingly, the assessment of loneliness alongside conventional symptom-based measures may provide additional clinically relevant information on psychosocial vulnerability and, in turn, help identify potentially modifiable targets for preventive and early-intervention strategies (Contribution 1).

The importance of interpersonal functioning is also evident at earlier developmental stages. Indeed, in one of the contributions to this Special Issue, emotion recognition ability was examined in preschool children and was found to be associated with socioemotional adjustment (Contribution 2). More in detail, changes in emotion recognition were observed over time among children participating in a socioemotional intervention; however, the study design did not allow these changes to be causally attributed to the intervention (Contribution 2). Importantly, lower recognition accuracy was associated with greater socioemotional difficulties, particularly peer-related problems (Contribution 2). These findings support the relevance of emotion recognition to social functioning, while highlighting the need for caution in interpreting the direction of this association [8]. The distinction between specific perceptual mechanisms underlying emotion recognition and broader dimensions of socioemotional competence has important methodological implications, as global behavioural measures may not adequately capture the processes involved in decoding emotional signals (Contribution 2) [9]. Longitudinal studies integrating behavioural and neurobiological measures may therefore help clarify how these processes evolve and relate to social adjustment over development (Contribution 2).

A second major area addressed by this Special Issue concerns the role of digital environments in shaping specific dimensions of mental health among young adults (Contribution 3). Indeed, evidence from one study suggests that the use of specific social media platforms may be associated with distinct patterns of vulnerability related to feeding and eating disorders and body image dissatisfaction (Contribution 3). In particular, TikTok use was associated with greater eating disorder risk and body image concerns, whereas Instagram use was more specifically associated with body dissatisfaction (Contribution 3). Moreover, problematic social media use and engagement with specific types of online content, including body positivity and body neutrality content, as well as mukbang-related videos, a form of online content featuring individuals consuming large quantities of food on camera, were associated with greater eating disorder vulnerability, although the cross-sectional design precludes causal interpretation (Contribution 3). Taken together, these findings highlight the complex relationship between social media use and psychological vulnerability, which appears to depend not only on time spent online but also on platform characteristics, content exposure, and appearance-based social comparison [10]. A more comprehensive assessment of digital engagement may therefore improve understanding of eating- and body-image-related vulnerability [11]. At the same time, the widespread use of TikTok and Instagram among vulnerable young people suggests that these platforms may also represent strategic channels for delivering psychoeducational, media-literacy, and preventive interventions (Contribution 3). Digital environments may therefore be conceptualised not only as potential contexts of vulnerability but also as opportunities for targeted prevention and the dissemination of evidence-based health information [12].

The integration of engaging approaches tailored to young people’s developmental needs, together with digital innovation, is further reflected in an intervention based on Role Playing Games (RPGs) for adolescents and young adults with anxiety, depressive symptoms, and emotional dysregulation (Contribution 4). The proposed programme combines collaborative narrative role-playing with metacognitive and mentalisation processes guided by clinicians, addressing symptoms, emotion regulation, and coping, as well as broader abilities such as theory of mind and self-reflectiveness (Contribution 4). RPG-based interventions may be particularly relevant to youth mental health, as they embed therapeutic processes within engaging formats that may enhance motivation, participation, and treatment adherence [13]. Moreover, their delivery in both face-to-face and online settings may increase accessibility and flexibility, while potentially reducing logistical and stigma-related barriers commonly associated with conventional mental health services [14]. Nevertheless, as the available evidence remains preliminary, rigorous evaluation of efficacy, acceptability, feasibility, and longer-term outcomes is required before broader implementation in routine clinical practice (Contribution 4).

Finally, the importance of clinical heterogeneity is further highlighted by evidence on sleep quality among young individuals with eating disorders (Contribution 5). Indeed, a person-centred latent profile analysis identified four distinct patterns of sleep functioning, which differed in eating psychopathology, affective symptoms, emotion regulation difficulties, and sleep-related eating behaviours. Among the clinical variables examined, night eating showed the strongest and most consistent association with the more severely impaired sleep profiles (Contribution 5). Taken together, these findings highlight substantial clinical heterogeneity within conventional diagnostic categories, with sleep disturbances potentially both reflecting and maintaining eating disorder psychopathology (Contribution 5). Accordingly, tailored approaches may complement traditional categorical frameworks by identifying clinically meaningful subgroups and informing more personalised approaches to assessment and intervention [15].

## 3. Toward a Developmental and Precision Framework for Youth Mental Health

Rather than identifying a single emerging risk factor or therapeutic target, the contributions to this Special Issue support a broader view of youth mental health based on dynamic processes that extend beyond conventional diagnostic boundaries [2,3]. From childhood through adolescence and into early adulthood, vulnerability appears to reflect the interaction between individual susceptibility, interpersonal functioning, developmental stage, and environmental exposures [2,3]. In this framework, loneliness, emotion processing difficulties, sleep disturbances, and problematic digital engagement may represent clinically relevant dimensions whose significance varies according to developmental and clinical context (Contributions 1–5) [3].

The heterogeneity observed within conventional diagnostic categories further supports dimensional and person-centred models (Contribution 5) [15]. Indeed, differences in symptom expression and associated clinical features may help identify subgroups characterised by distinct vulnerabilities and therapeutic needs, consistent with a precision mental health framework (Contribution 5) [15].

Digital environments add further complexity, as their effects may depend on platform characteristics, content exposure, patterns of engagement, and individual vulnerability (Contribution 3) [11,12]. The same perspective applies to digital and game-based therapeutic approaches, whose clinical relevance should be evaluated in relation not only to symptom improvement but also to engagement, accessibility, and suitability for younger populations [13,14] (Contribution 4).

From a methodological perspective, this framework requires moving beyond cross-sectional associations and average group effects (Contributions 2,3). Longitudinal designs may help clarify temporal ordering and prospective associations among risk factors and psychopathological outcomes, while the integration of behavioural and neurobiological measures may provide a more dynamic characterisation of developmental trajectories (Contribution 2). The evaluation of therapeutic interventions should likewise extend beyond efficacy to include acceptability, accessibility, persistence of effects, and variability in treatment response [13,14] (Contribution 4). Advancing youth mental health may therefore require translating this increasingly differentiated understanding of vulnerability into more individualised approaches to prevention and care.

## 4. Conclusions

Youth mental health reflects the complex interplay between developmental processes, individual experience, and the social environment. In this line, the contributions to this Special Issue support approaches that move beyond rigid diagnostic categories and better account for the heterogeneity of clinical presentations across development. Future research should clarify how vulnerability and resilience evolve over time and translate this knowledge into effective and accessible interventions suited to young people’s needs.
